# Appropriate criteria for identification of near-miss maternal morbidity in tertiary care facilities: A cross sectional study

**DOI:** 10.1186/1471-2393-7-20

**Published:** 2007-09-11

**Authors:** JP Souza, JG Cecatti, MA Parpinelli, SJ Serruya, E Amaral

**Affiliations:** 1 Department of Obstetrics and Gynecology, University of Campinas, Campinas, São Paulo, Brazil; 2 Department of Science and Technology, Ministry of Health, Brasília, DF, Brazil

## Abstract

**Background:**

The study of severe maternal morbidity survivors (near miss) may be an alternative or a complement to the study of maternal death events as a health care indicator. However, there is still controversy regarding the criteria for identification of near-miss maternal morbidity. This study aimed to characterize the near miss maternal morbidity according to different sets of criteria.

**Methods:**

A descriptive study in a tertiary center including 2,929 women who delivered there between July 2003 and June 2004. Possible cases of near miss were daily screened by checking different sets of criteria proposed elsewhere. The main outcome measures were: rate of near miss and its primary determinant factors, criteria for its identification, total hospital stay, ICU stay, and number and kind of special procedures performed.

**Results:**

There were two maternal deaths and 124 cases of near miss were identified, with 102 of them admitted to the ICU (80.9%). Among the 126 special procedures performed, the most frequent were central venous access, echocardiography and invasive mechanical ventilation. The mean hospital stay was 10.3 (± 13.24) days. Hospital stay and the number of special procedures performed were significantly higher when the organ dysfunction based criteria were applied.

**Conclusion:**

The adoption of a two level screening strategy may lead to the development of a consistent severe maternal morbidity surveillance system but further research is needed before worldwide near miss criteria can be assumed.

## Background

The World Health Organization (WHO) estimated that, in the year 2000, 20 million women suffered acute complications in pregnancy with the occurrence of 529,000 maternal deaths [1-3]. In Brazil, the official estimate of maternal mortality ratio is approximately 73 cases for every 100,000 liveborn infants, although according to the WHO, this ratio may be as high as 260 cases per 100,000 liveborn infants [1,4]. The disparity between the official figures and the WHO estimate reflects the difficulty in calculating and characterizing the profile of maternal mortality in Brazil. This difficulty is attributed to the incomplete vital registries, to the under-notification of causes of maternal death and to the dispersion of the cases over the wide geographical area involved [5]. These same factors are believed to contribute worldwide to the negligence of the problem of maternal mortality, and this has stimulated specialists to seek new indicators capable of contributing more effectively towards its combat [2]. Maternal mortality in developed countries is an infrequent event that is becoming even rarer, and for this reason interest in severe obstetrical morbidity, and specifically in cases referred to as near miss, has increased.

Cases of *near miss *are those in which women present potentially fatal complications during pregnancy, delivery or during the puerperium, and who survive merely by chance or by good hospital care. Near miss cases occur more often than maternal death and may generate more information because the woman herself can be a source of data. Once severe maternal morbidity precedes maternal death, the systematic identification and the study of near miss cases may provide further understanding of the determinants of maternal mortality [6,7].

The study of near miss cases has also been used to evaluate the quality of obstetrical care, leading to improved understanding of cases of maternal death, since survival in cases of near miss occurs mainly because of the care available [7]. Indeed, women with severe morbidity are frequently transferred to clinical or surgical intensive care units, although no specific consensus has yet been defined regarding the intensive care of these women [8]. Within this context, the lack of planning with regard to this type of care may delay the implementation of necessary measures and this delay has been associated with unfavorable maternal-fetal outcomes [9].

It is therefore understandable why the insertion of the near miss concept in the strategy for the combat of maternal death seems to be a justifiable action. Indeed, this is a recent and still slightly abstract concept that has been widely used by different authors, generating a certain degree of controversy regarding its definition [7,10]. Three different kinds of definitions have been used to describe near miss maternal morbidity: the definitions based on the admission of women to intensive care units during the pregnancy-puerperium cycle [11-14]; those based on the occurrence of certain diseases or complications such as preeclampsia, hemorrhage or severe sepsis [15-17] and those based on evidence of organic dysfunction [18].

Nevertheless, a direct comparison of these current definitions is still not feasible at all, once that there is no worldwide recognized gold standard available to identify near miss. In this context, it has not yet been formally tested if the cases identified as near misses would be different or the same using different definitions. To address this point and considering the concept existing behind the actual proposed definitions of near miss, we attempted to evaluate the closeness of these definitions to the original concept. We compared the complexity of care required, in an assumption where the near miss cases would be those more severe and the more severe cases would require more interventions. We also evaluated the primary determinants of severe morbidity, the maternal-fetal outcome, and the incidence of near miss according to each definition, over a period of one year, in a tertiary reference center for women's health.

## Methods

A descriptive study was carried out between 1^st ^July 2003 and 30^th ^June 2004 at the maternity hospital of the University of Campinas, the teaching hospital of a public university that provides tertiary care to a region of approximately three million inhabitants in the municipality of Campinas in the state of São Paulo, Brazil. This hospital is equipped with surgical theaters, a blood bank and an obstetrical and neonatal intensive care unit. Teams of obstetricians, anesthesiologists, neonatologists and intensive care specialists are available round the clock. Specialists of other clinical or surgical fields working in other units of the university hospital complex are readily available for consultation. The present study received the approval of the Institutional Review Board prior to initiation.

A specific operational definition of severe maternal morbidity was adopted for this study. According to this definition, every woman with any clinical condition compatible with any criteria established by Mantel et al. [18] or Waterstone et al. [17] to define severe morbidity during gestation, delivery or in the first 42 days of puerperium was classified as a case of near miss (Table 2).

**Table 2:** Criteria used to initially identify and classify cases of near miss

**Waterstone's Criteria**	**N**	**%**
Severe preeclampsia	45	36.3
Eclampsia	12	9.7
HELLP syndrome	7	5.6
Severe hemorrhage	13	10.5
Severe sepsis	8	6.4
Uterine rupture	1	0.8
Sub-total	86	69.3

**Mantel's Criteria**	**N**	**%**

Admission to the ICU for whatever reasons	40	32.3
Hypovolemia requiring 5 or more units of packed red blood cells	9	7.3
Pulmonary edema	5	4.0
Emergency hysterectomy for any reason	4	3.2
Admission to the ICU for sepsis	1	0.8
Intubation and ventilation for more than 60 minutes except for general anesthesia	1	0.8
Diabetic ketoacidosis	1	0.8
Coma for more than 12 hours	1	0.8
Cardio-respiratory arrest	-	-
Peripheral O_2 _saturation <90% for more than 60 minutes	-	-
Ratio Pa O_2_/FiO_2 _< 300 mmHg	-	-
Oliguria, defined as diurese <400 ml/24 h, refractory to careful hydration or to furosemide or dopamine	-	-
Acute urea deterioration to 15 mmol/l or creatinine >400 mmol/l	-	-
Jaundice with preeclampsia	-	-
Thyrotoxic crisis	-	-
Acute thrombocytopenia requiring transfusion of platelets	-	-
Sub-arachnoid or intra-parenchymatous hemorrhage	-	-
Anesthetic accident: (1) severe hypotension associated with epidural or rachidian anesthesia – hypotension defined as systolic pressure <90 mmHg for more than 60 minutes; (2) failure in tracheal intubation requiring anesthetic reversion	-	-
Sub-total	62	50.0

The identification and classification of cases of severe maternal morbidity was carried out by means of a daily visit to the Delivery Room, the Intensive Care Unit and to the Ward of Obstetrical Pathology of the institution, in the same way described by Mantel et al. [18]. Whenever a woman was identified as a case of severe maternal morbidity, the criteria initially recognized were considered as the criteria for inclusion in the study and if a woman was included by presence of criteria defined by Mantel, the coexistence of criteria defined by Waterstone was also studied, and vice-versa. The chart review was performed after the women's hospital discharge. On discharge, the presence of further criteria of severe morbidity was assessed. The staff responsible for the woman's hospital care was not told that the woman had been identified as a near miss case in the study in order to avoid possible biases in conduct.

The following data were collected in all cases: age, gestational age at the time of classification as near miss and at the end of pregnancy, time of puerperium at the moment of classification as near miss, parity, previous Caesarian section, previous abortion, type of delivery, previous morbidity, morbidity during pregnancy, primary determinant factor of severe morbidity (in an analogy to the basic cause of maternal death), criteria indicative of severe morbidity according to Mantel et al. [18], and criteria indicative of severe morbidity according to Waterstone et al. [17]. Information was also collected on admission to the intensive care unit, including the reason for admission to the ICU (monitoring and surveillance or intensive care) and duration of stay in the ICU. Information relating to the use of blood-derivatives and special procedures was also collected. Special procedures were defined as propedeutic or therapeutic interventions not normally used during prenatal care, delivery or during the puerperium, e.g. central venous access, insertion of an arterial line, invasive hemodynamic monitoring, echocardiograph, non-obstetrical ultrasonography, hysterectomy, mechanical ventilation, among others). The total duration of hospital stay, occurrence of organ dysfunction, condition at discharge from hospital and perinatal results were also evaluated.

These data were collected on a pre-coded form specially developed for this purpose. After resolution of each case, the consistency and availability of the information on the form was reviewed by one of the investigators and this data was inserted into a database using Microsoft Excel 2003 software. Subgroups of women were identified according to the set of criteria for near miss used for their classification as such. Each subgroup was compared with the remainder of the sample with respect to the total time of hospital stay and number of special procedures carried out.

## Results

During the twelve months of the study, 2,929 women were delivered at the institution and 124 women were classified as severe maternal morbidity, according to the mixed set of criteria applied. Two maternal deaths and 81 fetal deaths occurred during the same period, resulting in a ratio of maternal death of 70.1 maternal deaths per 100,000 live births and a stillbirth rate of 27.6 per 1,000 deliveries. The rate of maternal death in severe morbidity was 1.6%, what means that the case/fatality ratio was 62:1.

The characteristics of the women classified as near miss were: mean age 27.5 (± 7.4) years (slightly more than 15% of the women were adolescents), 42 women were nulliparous and 32 had had three or more previous deliveries. Ninety-nine women were pregnant and the other 25 were admitted to the study during the puerperium. Mean gestational age at the time of inclusion to the study was 30.3 (± 6.9) weeks and 90% of the women in the puerperium were in the first week following delivery. When comparing with data from the general population attending the same maternity, the most marked differences of near miss cases were the earlier interruption of pregnancy and the higher rate of Caesarean section (Table 1). Considering the two groups of cases of maternal morbidities (Mantel and Waterstone cases), no differences were observed (data not shown).

**Table 1:** Characteristics of near miss cases and general obstetric population (GOP) attending the tertiary care Maternity

	Near miss cases		GOP
		
Characteristics	N	%	%
Age (n = 124)			
<20 years	19	15.3	7.3
20–29 years	52	41.9	54.2
30–39 years	48	38.7	34.0
>39 years	5	4.1	4.5
			
Parity (n = 124)			
0	42	33.9	36.5
1–2	50	40.3	41.6
3 or more	32	25.8	21.9
			
Resolution of pregnancy at (n = 118):			
Gestational age <28 weeks	16	13.6	0.3
Gestational age between 28 and 32 weeks	25	21.2	1.4
Gestational age between 33 and 36 weeks	35	29.6	5.9
Gestational age > 36 weeks	42	35.6	92.4
			
Type of delivery (n = 118)			
Vaginal	21	17.8	66.1
Caesarian	97	82.2	33.9
			
Abortions	4	3.2	NA
Stillbirths	12	11.2	NA
Use of neonatal ICU	39	36.4	NA
			
Primary determinant factors of near miss			
Hypertensive syndromes	71	57.3	--
Hemorrhage	17	13.7	--
Sepsis	6	4.8	--
Abortion	4	3.2	--
Non-obstetrical complication	26	21.0	--

NA: Not available

Four cases of near miss occurred due to miscarriage and three of those women were discharged from hospital in good condition prior to resolution of the gestation and were referred to their primary care center. One of these women became lost-to-follow-up and there is no data on the outcome of her pregnancy. Of a total of 118 deliveries, there were 21 vaginal deliveries (two requiring the use of forceps) and 97 Caesarian sections (82.2%). Mean gestational age at the time of resolution of the pregnancy was 33.1 (± 5.9) weeks.

Fifty-seven women (46%) had at least one significant morbid antecedent and the most frequent cause of morbidity was preeclampsia, which was present in 64 women (51.2%). The primary determinant factors of severe morbidity detected in this sample, as well as other general characteristics of the samples, are in Table 1. In more than half the cases, hypertensive syndromes were the primary determinant factor of near miss.

With reference to the criteria used to classify near miss, 62 women were initially included according to Mantel's proposal, while 86 were classified according to Waterstone's proposal. Twenty-four women presented criteria common to both authors at the moment of inclusion as a case of near miss. Of the six criteria proposed by Waterstone, all were used at least once in the present sample, while only 8 of the 19 criteria proposed by Mantel were used, as shown in Table 2. Severe preeclampsia and admission to the ICU were the main causes of near miss found in 45 and 40 women respectively. Other frequent causes were severe hemorrhage (13 women) and eclampsia (12 women).

During the hospital stay, 49 women initially classified by Waterstone's criteria were sent to ICU. At the hospital discharge, these women could also be classified as severe maternal morbidity according to Mantel criteria. The number of patients that anytime during their hospital stay would have qualified for any definition is 78. At any time during hospital stay, 112 women would fit the Mantel definition and 90 women would fit the Waterstone definition. The 34 women not incorporated by the Waterstone definition presented heart disease (15:34), respiratory complications (4:34), other non obstetric complications (13:34). They also included a case of ectopic pregnancy and a case of abruptio placentae that could not be classified by Waterstone as severe hemorrhage but has been managed in the ICU. Twelve women not incorporated by the Mantel definition presented severe preeclampsia.

Regarding the ICU utilization, a total of 112 women were admitted to the ICU, 35 of these for intensive clinical support and the others for monitoring and surveillance. However, only 40 women were initially included in the study because of the criterion "admission to ICU". The median duration of stay in the ICU was 3 days (range 1–50 days) and 15 women were submitted to invasive mechanical ventilation for at least one day, for a total of 67 days of artificial ventilation. Thirty-one women required a transfusion of blood-derivatives and used 144 units of packed red blood cells, 82 units of fresh frozen plasma, 36 units of concentrated platelets and 11 units of cryoprecipitate. Of this total, nine women received five or more units of packed red blood cells and were included in the study on the basis of the respective criterion established by Mantel, as shown in Table 2. A total of 45 women developed organ dysfunction and all of them had been admitted to the ICU.

The severe maternal morbidity ratio varied between 15 cases/1000 deliveries and 42 cases/1000 deliveries, according to the definitions used: mixed criteria – 42 cases per 1000 deliveries (124:2929), Mantel – 38/1000 deliveries (112:2929), Waterstone – 31/1000 deliveries (90:2929), ICU utilization – 38/1000 (112:2929) and organ dysfunction – 15/1000 deliveries (45:2929).

Forty-five women (36.3%) required special procedures and a total of 126 special procedures were performed, 102 of them in women admitted to the ICU for intensive support (80.9%). The most frequent procedures carried out were the installation of central venous access, echocardiograph and invasive artificial ventilation, as shown in Table 3. Although eight women were submitted to hysterectomy, in only four of them was this procedure the initial criteria for classification as *near miss*.

**Table 3:** Special procedures carried out in the care of women with near miss

Procedures	N	%
Central venous access	21	16.6
Arterial access/invasive arterial pressure	10	7.9
Invasive artificial ventilation	15	11.9
Non-invasive artificial ventilation	4	3.2
Invasive hemodynamic monitoring	2	1.6
Hemodynamic support with vasoactive drugs	11	8.7
Tomography	11	8.7
Echocardiography	17	13.5
Other non-obstetrical echography	13	10.3
Hysterectomy	8	6.3
Other special procedures*	14	11.1

*emergency hemodialysis, electric cardioversion, digestive endoscopy, electroneuromyography, etc.

The mean total duration of hospital stay was 10.3 (± 13.24) days. Table 4 shows the mean total duration of hospital stay and the number of procedures by subgroups of women classified according to the criteria proposed by Mantel or Waterstone, or according to admission to the ICU for whatever cause or for intensive support, compared to the rest of the sample. In general, the duration of hospital stay and the number of special procedures were significantly greater when Mantel's criteria or admission to the ICU for intensive support was used.

**Table 4:** Total duration of hospital stay (in days) and number of special procedures in the care of women with near miss, by subgroups

Sub-group	Duration of hospital stay			Special procedures		
	Criteria present	Criteria absent	p	Criteria present	Criteria absent	p

Waterstone ^17^	9.7 ± 11.1	11.7 ± 17.2	0.43	0.9 ± 1.99	1.2 ± 1.7	0.45
Admission to ICU	10.7 ± 13.8	6.2 ± 3.1	0.26	1.2 ± 1.98	-	0.04
Mantel ^18^	12.6 ± 17.9	7.9 ± 4.2	0.04	1.7 ± 2.3	0.3 ± 0.9	<0.001
Admission to ICU for intensive care	16.5 ± 22.9	7.8 ± 4.6	<0.001	2.9 ± 2.6	0.3 ± 0.7	<0.001

*emergency hemodialysis, electric cardioversion, digestive endoscopy, electroneuromyography, etc.

Regarding maternal-fetal outcome, 116 women were discharged from hospital in conditions of good health, whereas 8 women were discharged with at least one sequela (8 cases of infertility because of hysterectomy and, in one of these women, poly neuromyopathy associated with sepsis. Two women were excluded from the analysis because they died. The first of these cases was a 21-year old pregnant woman, primigravida, who had cardiac insufficiency and severe pulmonary hypertension secondary to valvular cardiopathy (double mitral lesion, tricuspid and aortic). Approximately one week prior to her death, this pregnant woman's cardiac insufficiency became progressively worse. At 28 weeks of gestational age, she had been referred from another tertiary care hospital to the ICU, where maternal and fetal death occurred on the day of her admission following sudden accentuated hemodynamic deterioration. The second case of maternal death also occurred in a 21-year old woman who had had one previous delivery and who had been hospitalized one week earlier in a secondary hospital with jaundice during the 35^th ^week of gestation. After four days of hospitalization, she was found to have premature placental abruption, and subsequent fetal death occurred. She then developed a coagulopathy and was referred to the ICU where she arrived with multiple organ dysfunction and died four days later. Both cases of maternal death, however, were initially identified as near miss according to Mantel's criteria of "admission to the ICU for whatever cause".

With respect to fetal outcome, 12 stillbirths occurred (five following vaginal delivery and seven following Caesarian section), resulting in a stillbirth rate four times higher than that observed in the hospital (112.1 stillbirths for every 1,000 deliveries).

## Discussion

The severe maternal morbidity ratio ranged between 15 and 42 cases per 1,000 deliveries, depending on the set of criteria used. These ratios are located within the wide range of ratios of near miss described in the literature (0.7 – 119.9 per 1,000 deliveries) [10]. On the other hand, the maternal mortality ratio, which is used to help evaluate the quality of care offered and to permit a comparison of the performance of different services [19], was low compared to that observed in developing countries [10]. However, it should be taken into consideration that this ratio is greatly influenced by the definition of near miss adopted and that indicators of the quality of the therapeutic process in general should consider the degree of clinical severity, which does not occur when the present indicator is used.

Although undesirable, high rates of Caesarian section may be acceptable among women who develop severe maternal morbidity due to the urgency required to resolve the gestation and the factors that may make a vaginal delivery difficult to occur. On the other hand, it is known that in Brazil, even in the general population, the Caesarian section rate is high, which in itself contributes to it being performed in subsequent gestations [20]. In studies among women admitted to intensive care units in Brazil, the observed Caesarian section rates were 72.4% and 75.5% [13,14]. These rates are similar to those reported in the international literature by different investigators for women submitted to intensive care [11,15]. In the present study, the incidence of Caesarian sections was high (82.2%) and it is possible that the principal determinant of this rate was the severe morbidity in itself, since the rate of Caesarian sections was significantly higher in the population of women who developed severe morbidity during pregnancy compared to those who developed it during the puerperium.

With reference to the primary determinant factors of severe morbidity, it was observed that hypertension occupied the main role, and this is in agreement with its position as the basic cause of maternal death most often found in Brazil [4]. Non-obstetrical complications and hemorrhages were the other most common primary determinant factors of severe morbidity.

Despite the high ratios of maternal mortality in Brazil, maternal death tends to be a relatively rare event in Brazilian hospitals. In this study, two cases of maternal death occurred, both in patients referred from other institutions, while 124 women developed severe morbidity. Considering the present sample, the absolute majority of severe and potentially fatal obstetrical complications observed would have been excluded from a more profound analysis and the two cases of death certainly do not constitute a representative sample of the 124 cases. In this respect, it is possible that the evaluation of the risk factors eventually identified in the two cases of death may contribute little to the discussion on strategies to reduce the potential risk of maternal death in the 124 cases that developed severe morbidity. Even considering that only one institution was evaluated and that it could represent a bias, there is no concrete reason to suppose the situation would be different for the whole population.

Maternal hospitalization has permitted evaluations of complications during pregnancy [21]. In this way, in studies of critically ill individuals, clinical severity has been correlated with the complexity of care, quantifying, for example, the degree of therapeutic intervention [22]. In the present study, it was possible to identify a tendency following which the women who were initially classified as near miss according to Waterstone's criteria presented a shorter duration of hospital stay and a lower demand for therapeutical interventions, whereas the women who were classified according to Mantel's criteria presented a tendency to require longer hospitalization and a greater number of therapeutic interventions. The criteria proposed by Mantel are based on the occurrence of organic dysfunction or on the greater complexity of management and, although they may be more precise, the application of these criteria is linked to the use of more complex resources, such as laboratory tests and the use of intensive care, which may delay the identification of cases and limit the applicability of the criteria.

Several studies adopted definitions of near miss based on the admission of women to intensive care units [10]. Admission to the ICU may represent the most severe cases; however, depending on the availability of beds and on their accessibility, the degree of gravidity may vary. In a large proportion of maternity hospitals, there are no ICU beds. Therefore, a large number of severe cases would be excluded from this definition and in many situations only the most severe cases would be transferred, resulting indeed in an increase in the specificity of cases but at the cost of a reduction in sensitivity. On the other hand, facilities that have an ICU tend to be more liberal in the use of their ICU beds with a reasonable percentage of women being admitted for monitoring and surveillance. In the present study, only a third of women admitted to the ICU were admitted for intensive support. Moreover, this was the group of women in whom more severe conditions developed. As a result of the significant number of women admitted to the ICU for monitoring and surveillance, it is possible that the inclusion of women according to Mantel's criteria has suffered a bias, leading to some over-estimation.

Analyzing the profile of the therapeutic interventions required for the care of women with severe morbidity, it can be observed that the majority of these women did not require procedures that would be considered special. More than 80% of all the special procedures were carried out in the 35 women submitted to intensive care, this group being the most complex and costly. In other words, the care of the majority of the women with severe maternal morbidity did not necessarily require expensive or highly complex resources and this fact should stimulate attitudes towards greater surveillance of patients at higher risk, since early detection of these cases permits more effective and possibly less complex interventions.

With respect to the concept of near miss, it is understood that in the cases that survive a potentially fatal complication during pregnancy, delivery or during the puerperium, this favorable outcome is a question of chance or of the implementation of good hospital care. It is therefore natural to suppose that a set of diagnostic criteria for near miss would be capable of identifying a population requiring greater attention and possibly more complex management, principally considering the association that exists between clinical severity and the complexity of care. Thus, if we start from the premise that the present study sample consists of a population that developed severe morbidity in the pregnancy-puerperium cycle, and consider the duration of hospital stay and the demand for special procedures as being indicative of the complexity of care, it can be concluded that the sub-group chosen according to the criteria proposed by Waterstone failed to identify a group that was significantly different from the other women with regards to the complexity of management. Waterstone criteria also leaves out several non obstetrical conditions (e.g. embolism, heart diseases). On the other hand, the criteria proposed by Mantel led to the selection of a group of women who received more complex care, and considering the linkage between complexity of care and severity, we believe that the Mantel set of criteria allowed the identification of a group of women closer to the concept of near miss than the Waterstone's criteria did.

Nevertheless, it is perhaps still premature to recommend the adoption of a certain set of criteria for the definition of severe morbidity, although it is possible to admit that carrying out a pre-selection with ample criteria of severe maternal morbidity, followed by a secondary selection with more rigid criteria such organ dysfunction or failure, may be more effective in identifying a group of women as near miss. A similar approach with results that seem promising was carried out by Geller et al. [23] in developing a severe morbidity score for the identification of near miss cases.

Indirectly, the results of the present study suggest that Mantel's criteria are probably better for screening cases of obstetrical near miss as being at risk of maternal death. However, as their use involves greater complexity with respect to the capabilities of clinical care installations, it would probably not be a good strategy to entirely discard criteria similar to those proposed by Waterstone in order to end up with a wider range of criteria that could be amply recommended and used in different contexts, including the reality of many developing countries. According to this, a mixed set of severe maternal morbidity criteria was used to evaluate the occurrence of severe maternal morbidities in Canada, using the diagnostic codes according to the International Classification of Diseases [24], as they are sometimes regularly registered in clinical records and in routine information system on health.

Nevertheless, reduction in the number of complications during pregnancy has long been the principal objective of various programs seeking to combat maternal death. However the efficacy of these interventions has been questioned and analyses over the past ten years have suggested that the majority of obstetrical complications are indeed unpredictable and not preventable [9]. On the other hand, the appropriate treatment for the immense majority of these complications is known and its adequate use would make the deaths that result from a large proportion of these complications avoidable. It is exactly on this point that the future perspective of a real system of epidemiological surveillance rests, based on the early identification of near miss cases, allowing the implementation of an adequate level of surveillance and care, with the theoretically consequent prevention of avoidable complications and deaths [25].

Another aspect that should not be forgotten concerns the reproductive future of women who survive severe complications during pregnancy, childbirth or during the puerperium. The occurrence of a near miss event during pregnancy may serve as an alert to the health services of a greater future reproductive risk and may perhaps differentiate the obstetrical care of this patient in subsequent pregnancies and intensify family planning services for these women.

## Conclusion

The study of severe maternal morbidity worldwide may provide a valuable contribution to the reduction of maternal mortality. In this way an effort should be directed for the development of a common set of criteria to allow the comparison of data. Additional studies are still needed, but the adoption of a two levels screening strategy may be appropriate. The first level should be based in comprehensive criteria of severe maternal morbidity, followed by the application of restrictive criteria such as organ dysfunction or failure. This approach may be effective for the development of a consistent severe maternal morbidity surveillance system.

## Competing interests

The author(s) declare that they have no competing interests.

## Authors' contributions

JGC, SJS and JPS conceived the study. All authors contributed to the design of the study. JPS carried out the severe maternal morbidity surveillance and data collection. JGC and MAP supervised the data collection. JPS and JGC performed the statistical analysis and wrote the first manuscript draft. All authors contributed to and approved the final manuscript.

## Pre-publication history

The pre-publication history for this paper can be accessed here:


